# An experimental design framework for Markovian gene regulatory networks under stationary control policy

**DOI:** 10.1186/s12918-018-0649-8

**Published:** 2018-12-21

**Authors:** Roozbeh Dehghannasiri, Mohammad Shahrokh Esfahani, Edward R. Dougherty

**Affiliations:** 1 Department of Biochemistry, Stanford University, CA, Stanford, 94305 USA; 2 Division of Oncology, Stanford School of Medicine, Stanford, 94305 CA USA; 3 Department of Electrical and Computer Engineering, Texas A&M University, College Station, 77843 TX USA; 4 Center for Bioinformatics and Genomic Systems Engineering, Texas A&M University, College Station, 77845 TX USA

**Keywords:** Experimental design, Gene regulatory networks, Mean objective cost of uncertainty (MOCU), Network intervention, Markov chains, Dynamical intervention

## Abstract

**Background:**

A fundamental problem for translational genomics is to find optimal therapies based on gene regulatory intervention. Dynamic intervention involves a control policy that optimally reduces a cost function based on phenotype by externally altering the state of the network over time. When a gene regulatory network (GRN) model is fully known, the problem is addressed using classical dynamic programming based on the Markov chain associated with the network. When the network is uncertain, a Bayesian framework can be applied, where policy optimality is with respect to both the dynamical objective and the uncertainty, as characterized by a prior distribution. In the presence of uncertainty, it is of great practical interest to develop an experimental design strategy and thereby select experiments that optimally reduce a measure of uncertainty.

**Results:**

In this paper, we employ mean objective cost of uncertainty (MOCU), which quantifies uncertainty based on the degree to which uncertainty degrades the operational objective, that being the cost owing to undesirable phenotypes. We assume that a number of conditional probabilities characterizing regulatory relationships among genes are unknown in the Markovian GRN. In sum, there is a prior distribution which can be updated to a posterior distribution by observing a regulatory trajectory, and an optimal control policy, known as an “intrinsically Bayesian robust” (IBR) policy. To obtain a better IBR policy, we select an experiment that minimizes the MOCU remaining after applying its output to the network. At this point, we can either stop and find the resulting IBR policy or proceed to determine more unknown conditional probabilities via regulatory observation and find the IBR policy from the resulting posterior distribution. For sequential experimental design this entire process is iterated. Owing to the computational complexity of experimental design, which requires computation of many potential IBR policies, we implement an approximate method utilizing mean first passage times (MFPTs) – but only in experimental design, the final policy being an IBR policy.

**Conclusions:**

Comprehensive performance analysis based on extensive simulations on synthetic and real GRNs demonstrate the efficacy of the proposed method, including the accuracy and computational advantage of the approximate MFPT-based design.

## Background

A salient aim of translational genomics is to develop new drugs via constructing gene regulatory network (GRN) models characterizing the interactions among genes and then use these models to design therapeutic interventions. Most intervention strategies in the literature assume perfect knowledge regarding the network model. However, unfortunately this is not a realistic assumption in many real-world biomedical applications as uncertainty is inherent in genomics due to the complexity of biological systems, experimental limitations, noise, etc. Presence of model uncertainty degrades the performance of interventions.

Markovian genetic networks, an example of which are probabilistic Boolean networks (PBNs), have received great attention in recent years [1–5]. These networks have been shown to be effective in mimicking the behavior of biological systems, particularly as they are able to capture the randomness of biological phenomena by means of a transition probability matrix (TPM). The long-run behavior of a Markovian network is determined by a steady-state distribution over network states. Designing therapeutic interventions for these networks, often studied in the context of Markov decision processes (MDPs), has been extensively studied over the past two decades [6]. The basic assumption behind many intervention algorithms is that the TPM is perfectly known.

When dealing with network models possessing uncertainty, it is prudent to design a robust intervention that provides acceptable performance across an *uncertainty class* of possible models compatible with the current state of knowledge. In general, the problem of designing robust operators (or interventions in this paper) is typically viewed from two different perspectives: minimax robustness and Bayesian robustness. Under a minimax criterion, the robust operator has the best worst-case performance across the uncertainty class. The main problem with minimax robustness is that it is very conservative and gives too much attention to outlier models in the uncertainty class that may possess negligible likelihood.

Bayesian robustness addresses this issue by assigning a prior probability distribution reflecting existing knowledge about the model. Under this criterion, the aim is to find a robust operator possessing the optimal performance on average relative to this prior distribution. In the context of Bayesian robustness, when optimality is relative to the prior distribution, the resulting operator is called an *intrinsically Bayesian robust* (IBR) operator, examples being IBR Kalman filter in signal estimation [7], IBR signal compression [8], and IBR network structural intervention for gene regulatory networks [9, 10]. When optimality is relative to the posterior distribution obtained by incorporating observations into the prior distribution, the robust operator is called an *optimal Bayesian operator* [11–14].

It is of prime interest to reduce model uncertainty via additional experiments and thereby improve the performance of the intervention. Since conducting all potential experiments is not feasible in many biomedical applications owing to operational constraints such as budget, time, and equipment limitations, it is imperative to utilize an *experimental design* strategy to rank experiments and then conduct only those experiments with high priority [15–18].

As experiments are aimed at reducing uncertainty, a crucial step in experimental design is uncertainty quantification. From a translational perspective, we are not concerned with overall uncertainty, but rather with the degradation induced by the uncertainty in the intervention performance. Taking this into account, we employ an objective-based uncertainty quantification scheme called the *mean objective cost of uncertainty* (MOCU) [10]. MOCU has been successfully used for developing experimental design in gene regulatory networks when structural interventions are concerned [15, 19–21].

In this paper, we extend the application of the objective-based experimental design for GRNs to the realm of dynamical interventions. The interactions among genes are characterized by a set of conditional probability matrices where the conditional probabilities in each matrix correspond to the regulatory relationship between a gene and its regulating genes. We address the experimental design problem involving a GRN model in which a number of probabilities across conditional probability matrices are missing. Unknown conditional probabilities are represented by conjugate prior distributions which are closed under consecutive observations. In this paper, we show how the uncertainty in the conditional probabilities can be translated into the uncertainty in an unknown transition probability matrix. Furthermore, we show how additional information in terms of a trajectory of consecutive state transitions from the true system, if available, can be integrated to update prior distributions to posterior distributions containing lesser uncertainty. Deriving IBR control policies, which involves minimizing the average cost relative to the prior distribution among all stationary control policies, is at the very core of our experimental design calculations. In this regard, we take advantage of the fact that an IBR control policy can be derived by using an *effective transition probability matrix* that represents the uncertainty class of transition probability matrices. We should emphasize the optimality of the IBR control policy, which is selected from all possible stationary policies as opposed to the model-constrained Bayesian robust (MCBR) control policy [22], which is selected from among only the policies that are optimal for networks belonging to the uncertainty class.

It is worth mentioning that due to the computational complexity limitation, we are only concerned with stationary control policies in this paper. Another approach for designing a Bayesian robust control policy is to design a non-stationary policy, referred to as the optimal Bayesian robust (OBR) control policy. In addition to the expected immediate cost and different future costs obtained due to being in different states at the next time steps, an OBR policy also considers the effect of observations obtained by different actions on the sequence of different posterior distributions, which makes an OBR policy be non-stationary. In an OBR setting, the control problem is transformed into an equivalent problem in which each state, being referred to as a hyperstate, contains both the ordinary state of the system and the state of the knowledge reflecting the prior information and the history of observations from the system. Utilizing the concept of hyperstates for designing OBR control policies has roots in the classical works of Bellman and Kalaba [23], Silver [24], Gozzolino [25], and Martin [26]. The major obstacle of the OBR theory is its enormous computational complexity [27–29], such that it cannot be applied to networks of larger than 4 genes, even when only network control is concerned [29], let alone experimental design whose complexity is several-fold more than that of the control problem. Hence, taking into account complexity considerations with OBR, we focus on IBR stationary policies for our experimental design problem, which still requires massive computations but at a more tolerable cost compared to OBR policies.

To mitigate the computational complexity burden of experimental design, and considering the fact that computing the IBR control policy can be computationally demanding, we approximate it by using the method of mean first passage time (MFPT) [30]. The main motivation behind utilizing MFPT for controlling GRN networks is the desire to reach desirable states and leave undesirable states in the shortest time possible. Using this intuition, MFPT is used in [31] to derive a stationary control policy that can be used as an approximation for the optimal control policy and in [32] to find the best control gene. Using the concept of MFPT, we approximate the IBR control policy required for the experimental design and thereby lower the complexity of the experimental design. We emphasize that the MFPT approximation is only used for experimental design and that the implemented control policy will always be the optimal stationary control policy.

We summarize the main contributions of the paper. (1) Despite all the previous MOCU-based experimental design methods whose focus was on structural interventions [15, 19, 20], in this paper we consider the class of stationary interventions and derive a closed-form solution for the IBR stationary intervention when the TPM is unknown. (2) While in the previous works, uncertain parameters involve a number of regulatory edges between genes, in this paper we consider the case that a number of conditional probabilities characterizing regulatory relationships between genes are unknown. Given that conditional probabilities can be estimated using time series gene expression data generated through a biological experiment, it is more realistic to consider these probabilities, rather than regulatory edges, as the outcomes of biological experiments. This new uncertainty assumption requires us to define a new uncertainty class and prior probability model. (3) To address the complexity concerns of the proposed method, we propose an approximate experimental design method utilizing mean first passage times (MFPTs) in which we extend the application of MFPT-based controls to unknown TPMs.

## Methods

### Markovian regulatory networks

In a network with *n* genes, a set of binary variables **V**={*X*_1_,*X*_2_,…,*X*_*n*_}, *X*_*i*_∈{0,1}, determines the expression states of the genes. The vector of gene expression values at time *t*, **X**(*t*)=(*X*_1_(*t*),…,*X*_*n*_(*t*)), referred to as the gene activity profile (GAP), defines the network state at each time step. In a Markovian regulatory network, network dynamics involves a trajectory of states over time governed by the transition rule **X**(*t*+1)=*f*(**X**(*t*),*w*(*t*)), *t*≥0, where *w*(*t*)∈*Ξ* captures randomness in the system and $f:\mathcal {S}\times \Xi \rightarrow \mathcal {S}$, $\mathcal {S}=\left \{0,1,\dots,2^{n}-1\right \}$ being the set of corresponding decimal representations for the network states, is a mapping that characterizes the state transitions in the network. The sequence of states over time can be viewed as a Markov chain characterized by a *transition probability matrix* (TPM) $\mathbf {P}=[P_{ij}]_{i,j=0}^{2^{n}-1}$, where *P*_*ij*_=Pr[**X**(*t*+1)=*j*|**X**(*t*)=*i*], Pr[ ·] being the probability operator. An ergodic Markov chain is guaranteed to possess a unique steady-state distribution **π**, such that **π**^*T*^=**π**^*T*^**P**, *T* being the transpose operator.

Assume that the expression state of a gene is solely determined by its regulating genes. In other words, given the values of its regulating genes, the expression state of a gene is conditionally independent from those of other genes. Let the vector of expression states of the regulating genes for *X*_*i*_ be denoted by $\Gamma _{X_{i}}$, where the ordering in $\Gamma _{X_{i}} $ is induced from the ordering *X*_1_,*X*_2_,…,*X*_*n*_. In a binary setting, if *X*_*i*_ has *k*_*i*_ regulating genes, then $\Gamma _{X_{i}}$ can have $2^{k_{i}}\phantom {\dot {i}}$ possible vector values. To define the regulatory relationship between gene *X*_*i*_ and its regulating genes, we can construct a *conditional probability matrix* (CPM) **C**(*X*_*i*_) of size $2^{k_{i}}\times 2\phantom {\dot {i}}$, where each row of the matrix corresponds to a certain combination of gene expressions in $\Gamma _{X_{i}}$ and the first and second columns correspond to the conditional probability of gene *X*_*i*_ being 0 and 1, respectively, i.e.,

1 $$\begin{array}{*{20}l} & C_{j,1}(X_{i})=\text{Pr}\left[X_{i}=0|\Gamma_{X_{i}}=j\right],  \\ & C_{j,2}(X_{i})=\text{Pr}\left[X_{i}=1|\Gamma_{X_{i}}=j\right], \end{array} $$

where by $\Gamma _{X_{i}}=j$ we mean that the equivalent decimal value of the vector of expression states for the regulating genes of *X*_*i*_ is *j*. A network of *n* genes with each gene having *k*_*i*_ regulating genes can be completely defined by *n* CPMs **C**(*X*_*i*_), 1≤*i*≤*n*, each being of size $2^{k_{i}}\times 2\phantom {\dot {i}}$. These matrices can be used to construct the transition probability matrix. Owing to the mutual conditional independence of all genes given the values of all regulating genes, the entry *P*_*ij*_ of the TPM can be found as

2 $$\begin{array}{*{20}l} P_{ij}& =\prod_{k=1}^{n}\text{Pr}\left[X_{k}=j_{k}\left|\bigcup\limits_{l=1}^{n}\right.\Gamma_{X_{l}}[i]\right] \\ & =\prod_{k=1}^{n}\text{Pr}\left[X_{k}=j_{k}\left|\Gamma_{X_{k}}\right.[i]\right] \notag \\ & =\prod_{k=1}^{n}C_{\Gamma_{X_{k}}[i],j_{k}+1}(X_{k}), \end{array} $$

where *j*_*k*_ is the binary value of the *k*-th gene in state *j* and $\Gamma _{X_{k}}[i]$ is the vector of binary values of the regulating genes for *X*_*k*_ extracted from the representation of state *i*. For example, consider a 3-gene network, *n*=3, in which gene *X*_1_ (*k*=1 in (2)) is regulated by genes *X*_2_ and *X*_3_. For this network, when computing *P*_14_ (*i*=1 and *j*=4 in (2)), *j*_*k*_=1 (as *X*_1_=1 for *j*=4) and $\Gamma _{X_{1}}[i]=(0,1)$ (as (*X*_2_,*X*_3_)=(0,1) for *i*=1).

From a translational perspective, the states of a network can be partitioned into two sets: desirable states $\mathcal {D}$, being associated with healthy phenotypes, and undesirable states $\mathcal {U}$, corresponding to pathological cell functions such as cancer. The goal of therapeutic interventions is to alter the dynamical behavior of the network in such a way as to reduce the steady-state probability $\pi _{\mathcal {U}}={\sum \nolimits }_{i\in \mathcal {U}}\pi _{i}$ of the network entering the undesirable states. There are two different approaches for network interventions: structural interventions and dynamical interventions. In a structural intervention, the goal is to modify the dynamical behavior of the network via a one-time change in its underlying regulatory structure [9, 33–35]. Dynamical interventions are typically studied in the framework of Markov decision processes and are characterized by control policies. These interventions usually involve the change in the expression of one or more genes, being called *control genes*, and can be applied either over a finite-time [36–38] or an infinite-time horizon [31, 39].

### Optimal dynamical control

Network interventions in this paper belong to the category of dynamical interventions. We assume that there is a control gene *g*∈**V** whose expression value is affected by a binary control input $c\in \mathcal {C}$, $\mathcal {C}=\{0,1\}$. The value of *g* is flipped when *c*=1 and not flipped when *c*=0. It is straightforward to extend the results to *m* control genes, where there are 2^*m*^ different control actions. Let $\mathbf {P}(c)=\left [P_{ij}(c)\right ]_{i,j=0}^{2^{n}-1}$ denote the controlled TPM, i.e.,

3 $$\begin{array}{*{20}l} P_{ij}(c)=\text{Pr}\left[\mathbf{X}(t+1)=j|\mathbf{X}(t)=i,c(t)=c\right]. \end{array} $$

The controlled TPM can be found using the uncontrolled TPM **P** as

4 $$\begin{array}{*{20}l} P_{ij}(c)=\left\{ \begin{array}{l} P_{ij}\qquad \qquad \text{if}\,\,c=0 \\ P_{\tilde{i}j}\qquad \qquad \text{if}\,\,c=1 \\ \end{array} \right.,  \end{array} $$

where states $\tilde {i}$ and *i* differ only in the value of gene *g*.

The problem of optimal control can be modeled as an optimal stochastic control problem [39]. Let the cost function $r(i,j,c):\mathcal {S}\times \mathcal {S}\times \mathcal {C}\rightarrow \mathbb {R}$ determine the immediate cost accrued when the network transitions from state *i* to state *j* under control action *c*. This cost reflects both the desirability of states and the cost for imposing control actions. Usually, larger cost values are assigned to the undesirable states and when the control action is applied. This cost function is assumed to be time-invariant, bounded, and nonnegative. We consider an infinite-horizon discounted cost approach as proposed in [39] in which a discount factor 0<*ζ*<1 is used to guarantee convergence [40]. Control actions are chosen over time according to a control policy *μ*=(*μ*_1_,*μ*_2_,…), $\mu _{t}:\mathcal {S}\rightarrow \mathcal {C}$. In this setting, given a policy *μ* and an initial state *X*_0_, the expected total cost is

5 $$\begin{array}{*{20}l} J_{\mu}(X_{0})={\lim}_{M\rightarrow \infty}\mathrm{E}\left[\sum_{t=0}^{M-1}\zeta^{t} r\left(\mathbf{X}(t),\mathbf{X}(t+1),\mu_{t}(\mathbf{X}(t)\right)\left|{\vphantom{\sum}} X_{0}\right.\right],  \end{array} $$

where the expectation is taken relative to the probability measure over the space of state and control action trajectories. If *Π* denotes the space of all admissible policies, we seek an optimal control policy *μ*^∗^(*X*_0_) such that

6 $$\begin{array}{*{20}l} \mu^{\ast}(X_{0})=\underset{}{\arg}\,\underset{\mu\in\Pi}{\min}\, J_{\mu}(X_{0})\qquad\qquad \forall X_{0}\in\mathcal{S}. \end{array} $$

The corresponding optimal expected total cost is denoted by *J*^∗^(*X*_0_). It has been shown that the optimal policy *μ*^∗^(*X*_0_) exists and can be found by solving *Bellman’s optimality equation* [40],

7 $$\begin{array}{*{20}l} J^{\ast}(i)\,=\,\underset{c\in\mathcal{C}}{\min}\left[\sum\limits_{j=0}^{2^{n}-1}P_{ij}(c)\left(r(i,j,c)+\zeta J^{\ast}(j)\right)\right]\quad\forall i\in\mathcal{S}. \end{array} $$

The optimal cost *J*^∗^ =(*J*^∗^(0),…,*J*^∗^(2^*n*^−1)) is the unique solution of (7) among all bounded functions and the control policy *μ*^∗^ that attains the minimum in (7) is stationary, i.e., *μ*^∗^=(*μ*^∗^,*μ*^∗^,..) [40]. In order to find the fixed point in the Bellman’s optimality equation and thereby find the optimal control policy, dynamic programming algorithms, including the value iteration algorithm that iteratively estimates the cost function, can be used.

### MOCU-based optimal experimental design framework

In this section, we review the general framework of the experimental design method in [15], which is based on the concept of the mean objective cost of uncertainty (MOCU) [10]. Let ***θ***=(*θ*_1_,*θ*_2_,…,*θ*_*T*_) be composed of *T* uncertain parameters in a network model. The set of all possible realizations for ***θ*** is denoted by *Θ* and is called an *uncertainty class*. A prior distribution *f*(***θ***) is assigned to ***θ***, which reflects the likelihood of each realization of ***θ*** being the true value.

For each possible intervention *ψ*∈*Ψ*, the class of interventions, and each model ***θ*** in the uncertainty class, an error function *ξ*_***θ***_(*ψ*) determines the error of *ψ* when applied to the network model ***θ***. The optimal intervention *ψ*(***θ***) has the lowest error relative to model ***θ***, i.e., *ξ*_***θ***_(*ψ*(***θ***))≤*ξ*_***θ***_(*ψ*),∀*ψ*∈*Ψ*. When dealing with an uncertainty class *Θ*, the intrinsically Bayesian robust (IBR) intervention *ψ*_IBR_(*Θ*) is defined as

8 $$\begin{array}{*{20}l} \psi_{\text{IBR}}(\Theta)=\underset{}{\arg}\,\underset{\psi\in\Psi}{\min}\,\mathrm{E}_{\boldsymbol{\theta}}\left[\xi_{\boldsymbol{\theta}}(\psi)\right],  \end{array} $$

where the expectation is taken relative to the prior distribution *f*(***θ***).

An IBR intervention is optimal on average rather than at each specific network model ***θ***; therefore, relative to ***θ*** an objective cost of uncertainty (OCU) can be defined as

9 $$\begin{array}{*{20}l} \mathrm{U}_{\Psi,\xi}(\boldsymbol{\theta})=\xi_{\boldsymbol{\theta}}(\psi_{\text{IBR}}(\Theta))-\xi_{\boldsymbol{\theta}}(\psi(\boldsymbol{\theta})). \end{array} $$

Taking the expectation of U_*Ψ*,*ξ*_(***θ***) relative to *f*(***θ***), we obtain the mean objective cost of uncertainty (MOCU):

10 $$\begin{array}{*{20}l} \mathrm{M}_{\Psi,\xi}(\Theta)&=\mathrm{E}_{\boldsymbol{\theta}}[\mathrm{U}_{\Psi,\xi}(\boldsymbol{\theta})]  \\ &=\mathrm{E}_{\boldsymbol{\theta}}[\xi_{\boldsymbol{\theta}}(\psi_{\text{IBR}}(\Theta))-\xi_{\boldsymbol{\theta}}(\psi(\boldsymbol{\theta}))].  \end{array} $$

MOCU measures the model uncertainty in terms of the expected increased error due to applying an IBR intervention (the chosen intervention in the presence of uncertainty) instead of an optimal intervention (the chosen intervention in the absence of uncertainty). Uncertainty quantification based on MOCU can lay the groundwork for objective-based experimental design.

Assume that corresponding to each parameter *θ*_*i*_, there is an experiment $\mathcal {E}_{i}$ that results in the exact determination of *θ*_*i*_. The goal of the experimental design is to find which experiment should be conducted first so that model uncertainty is reduced optimally. Focusing on experiment $\mathcal {E}_{i}$ and parameter *θ*_*i*_, consider the case that the outcome of experiment $\mathcal {E}_{i}$ is $\theta ^{\prime }_{i}$. Then the remaining MOCU given $\theta _{i}=\theta ^{\prime }_{i}$ is defined as

11 $$\begin{array}{*{20}l} &\mathrm{M}_{\Psi,\xi}\left(\Theta|\theta_{i}=\theta^{\prime}_{i}\right)  \\ &\qquad=\mathrm{E}_{\boldsymbol{\theta}|\theta^{\prime}_{i}}\left[\xi_{\boldsymbol{\theta}}\left(\psi_{\text{IBR}}\left(\Theta|\theta_{i}=\theta^{\prime}_{i}\right)\right)-\xi_{\boldsymbol{\theta}}\left(\psi\left(\boldsymbol{\theta}|\theta_{i}=\theta^{\prime}_{i}\right)\right)\right],  \end{array} $$

where the expectation is taken relative to the conditional distribution $f\left (\boldsymbol {\theta }|\theta _{i}=\theta ^{\prime }_{i}\right)$, $\Theta |\theta _{i}=\theta ^{\prime }_{i}$, is the reduced uncertainty class obtained after $\theta _{i}=\theta ^{\prime }_{i}$, and vector $\boldsymbol {\theta }|\theta _{i}=\theta ^{\prime }_{i}$ is obtained from vector ***θ*** by setting *θ*_*i*_ to $\theta _{i}^{\prime }$. Taking the expectation of (11) relative to the marginal distribution $f\left (\theta ^{\prime }_{i}\right)$, which is in fact the marginal distribution of the parameter *θ*_*i*_, we obtain the expected remaining MOCU given experiment $\mathcal {E}_{i}$ is carried out (or equivalently parameter *θ*_*i*_ is determined):

12 $$\begin{array}{*{20}l} {}&\mathrm{M}_{\Psi,\xi}(\Theta;\theta_{i})  \\ {}&=\mathrm{E}_{\theta^{\prime}_{i}}\left[\mathrm{M}_{\Psi,\xi}\left(\Theta|\theta_{i}=\theta^{\prime}_{i}\right)\right]  \\ {}&=\mathrm{E}_{\theta_{i}^{\prime}}\left[\mathrm{E}_{\boldsymbol{\theta}|\theta^{\prime}_{i}}\left[\xi_{\boldsymbol{\theta}}\left(\psi_{\text{IBR}}\left(\Theta|\theta_{i}=\theta^{\prime}_{i}\right)\right)-\xi_{\boldsymbol{\theta}}\left(\psi\left(\boldsymbol{\theta}|\theta_{i}=\theta^{\prime}_{i}\right)\right)\right]\right].  \end{array} $$

M_*Ψ*,*ξ*_(*Θ*;*θ*_*i*_) measures the pertinent uncertainty expected to remain in the model after conducting experiment $\mathcal {E}_{i}$. The experiment $\mathcal {E}_{i^{\ast }}\phantom {\dot {i}}$ that attains the minimum value of the expected reaming MOCU is called the *optimal experiment* and suggested as the first experiment [15]:

13 $$\begin{array}{*{20}l} i^{\ast}=\underset{}{\arg}\,\underset{i\in\{1,2,..,T\}}{\min}\,\mathrm{M}_{\Psi,\xi}(\Theta;\theta_{i}).  \end{array} $$

The parameter $\theta _{i^{\ast }}\phantom {\dot {i}}$ corresponding to $\phantom {\dot {i}}\mathcal {E}_{i^{\ast }}$ is called the *primary parameter*. Note that (13) can be further simplified through some mathematical manipulations and removing expressions not dependent on the optimization variable [20]:

14 $$\begin{array}{*{20}l} i^{\ast}=\underset{}{\arg}\,\underset{i\in\{1,2,..,T\}}{\min}\,\mathrm{E}_{\theta_{i}^{\prime}}\left[\mathrm{E}_{\boldsymbol{\theta}|\theta^{\prime}_{i}}\left[\xi_{\boldsymbol{\theta}}(\psi_{\text{IBR}}(\Theta|\theta_{i}=\theta^{\prime}_{i}))\right]\right].  \end{array} $$

A number of experimental design methods based on the MOCU framework have been proposed in the literature [15, 19, 20]. In all of these cases, the MOCU-based experimental design can reduce the number of needed experiments significantly in comparison to other selection policies such as entropy-based experimental design, pure exploitation, or random selection policy.

### Uncertainty in transition probability matrix

Assume that regulatory information between a gene and its regulating genes is missing for a number of genes in the network. In other words, a number of rows in the *n* conditional probability matrices are unknown. We represent unknown conditional probabilities by a set of random variables ***θ***=(*θ*_1_,*θ*_2_,…,*θ*_*T*_). Since each row of the CPM adds up to one, i.e., *C*_*j*,1_(*X*_*i*_)+*C*_*j*,2_(*X*_*i*_)=1, there is only one degree of freedom. The uncertainty in the CPMs will eventually show up in the corresponding TPM and thereby can affect the performance of the control policy. Therefore, it is of interest to reduce the uncertainty in the CPMs. We seek an experimental design method that efficiently guides us on which unknown conditional probability to determine first.

We need to assign prior distributions to the random variables representing unknown conditional probabilities. Assigning accurate priors is highly challenging. A prior distribution must describe the current state of knowledge regarding the unknown model accurately. It is also desirable that the prior distribution and the posterior distribution, obtained by incorporating data into the prior, belong to the same family of distributions, being referred to as a conjugate prior distribution. Using conjugate prior distributions, we can easily update the priors to the posteriors, which facilitates the computations in a Bayesian setting as it is enough to only keep track of the hyperparameters in the distributions. With this in mind, we utilize the beta distribution as the prior distribution for each unknown parameter *θ*_*i*_. Relative to a random variable *θ*_*i*_, the beta distribution Beta(*α*_*i*_,*β*_*i*_) with hyperparameters *α*_*i*_ and *β*_*i*_ is of the following form:

15 $$\begin{array}{*{20}l} \text{Beta}(\alpha_{i},\beta_{i})=\frac{\theta_{i}^{\alpha_{i}-1}(1-\theta_{i})^{\beta_{i}-1}}{B(\alpha_{i},\beta_{i})}, \end{array} $$

where *B*(*α*_*i*_,*β*_*i*_) is the beta function. The expected value of *θ*_*i*_ ∼ Beta(*α*_*i*_,*β*_*i*_) is $\mathrm {E}[\theta _{i}]=\frac {\alpha _{i}}{\alpha _{i}+\beta _{i}}$. When *α*_*i*_ = *β*_*i*_=1, the beta distribution becomes a uniform distribution over interval [0,1].

We assume that *θ*_1_,*θ*_2_,…,*θ*_*T*_ are independent and each parameter *θ*_*i*_, 1≤*i*≤*T*, has a beta distribution Beta(*α*_*i*_,*β*_*i*_); therefore, the prior distribution of ***θ***={*θ*_1_,*θ*_2_,…,*θ*_*T*_} is

16 $$\begin{array}{*{20}l} f(\boldsymbol{\theta})=\prod_{i=1}^{T}\text{Beta}(\alpha_{i},\beta_{i})\propto \prod_{i=1}^{T} \theta_{i}^{\alpha_{i}-1}(1-\theta_{i})^{\beta_{i}-1}. \end{array} $$

In addition to the set of CPMs, containing unknown conditional probabilities, it is possible that observations from network dynamics in terms of a trajectory $\mathcal {X}_{L}=\{\mathbf {X}(0),\mathbf {X}(1),\dots,\mathbf {X}(L)\}$ of *L* consecutive state transitions are also available. The state trajectory $\mathcal {X}_{L}$ can be utilized as an additional source of information to update the initial beta distributions to the posterior beta distributions. If *θ*_*i*_ represents the unknown conditional probability $C_{j,1}(X_{i})=\text {Pr}\left [X_{i}(t+1)=0|\Gamma _{X_{i}}=j\right ]$, then given a state trajectory $\mathcal {X}_{L}$ the posterior distribution $f(\theta _{i}|\mathcal {X}_{L})$ is again a beta distribution with new hyperparameters

17 $$\begin{array}{*{20}l} &\alpha^{\prime}_{i}=\alpha_{i}+\sum\limits_{l=0}^{L-1}\mathbbm{1}\left[\Gamma_{X_{i}}[\mathbf{X}(l)]=j,X_{i}(l+1)=0\right]  \end{array} $$

18 $$\begin{array}{*{20}l} &\beta^{\prime}_{i}=\beta_{i}+\sum\limits_{l=0}^{L-1}\mathbbm{1}\left[\Gamma_{X_{i}}[\mathbf{X}(l)]=j,X_{i}(l+1)=1\right],  \end{array} $$

where *X*_*i*_(*l*) denotes the value of gene *X*_*i*_ at the *l*-th state in the trajectory, and $\mathbbm {1}[\!\cdot ]$ is the indicator function. In other words, those state transitions in which the event corresponding to the unknown conditional probability *θ*_*i*_ occurs can be used to update the information about that unknown probability. Note that $\Gamma _{X_{i}}[\mathbf {X}(l)]=j$ implies that the equivalent decimal value of the gene expression vector for the regulating genes of *X*_*i*_ extracted from network state **X**(*l*) should be equal to *j*. The conditional expectation of *θ*_*i*_ given $\mathcal {X}_{L}$ is

19 $$\begin{array}{*{20}l} \mathrm{E}[\!\theta_{i}|\mathcal{X}_{L}]=\frac{\alpha^{\prime}_{i}}{\alpha^{\prime}_{i}+\beta^{\prime}_{i}}.  \end{array} $$

Since the uncertainty of an unknown conditional probability is governed by the corresponding terms *α*^′^, *β*^′^, and given the fact that observation(s) can potentially increase *α*’s and *β*’s according to (17) and (18), availability of a state trajectory $\mathcal {X}_{L}$ is equivalent to a lesser initial uncertainty, and hence a simpler experimental design problem.

### Optimal experimental design for determining unknown conditional probabilities

**Fig. 1:**
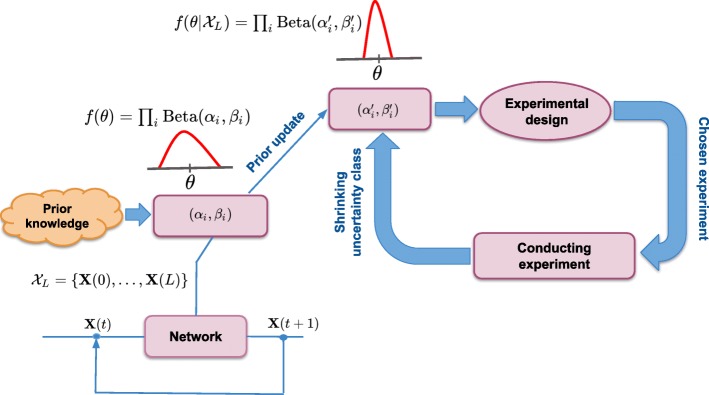
A schematic diagram of the proposed experimental design framework

Building on the general MOCU-based experimental design framework in (8)-(14), we propose an experimental design method when dynamical controls characterized by stationary control policies are concerned. A schematic diagram of the proposed experimental design framework is given in Fig. 1. We first assign beta distributions with initial hyperparameters (*α*_*i*_,*β*_*i*_) to each unknown conditional probability. Then if a state trajectory $\mathcal {X}_{L}$ is available as an additional source of knowledge, it is incorporated to update the initial hyperparameters to $\phantom {\dot {i}\!}\left (\alpha ^{\prime }_{i},\beta ^{\prime }_{i}\right)$ according to (17) and (18). These updated hyperparameters characterize the uncertainty class for finding the best parameter to determine using the proposed MOCU-based framework. When the first experiment is chosen and carried out, its outcome (the true value for the chosen unknown conditional probability) is incorporated in the uncertainty class, leading to a reduced uncertainty class that contains fewer uncertain parameters. If operational resources allow more experiments, this new uncertainty class can be used to find the next parameter for determination (this process can be iterated). Otherwise, the experimental design step is finished and the reduced uncertainty class is used to derive the IBR control policy based on which control actions at each time step are applied to the underlying true network.

As (14) suggests, in order to implement the experimental design, we need to derive IBR interventions. Therefore, we first focus on explaining how an IBR control policy can be derived. Considering an uncertainty class *Θ* of TPMs, relative to an initial state *X*_0_, the average expected total discounted cost across *Θ* for control policy *μ* = (*μ*_1_, *μ*_2_, …) is:

20 $$\begin{array}{*{20}l} &{}J^{\Theta}(\mu;X_{0})=\mathrm{E}_{\boldsymbol{\theta}}[J^{\boldsymbol{\theta}}(\mu;X_{0})]  \\ &{}=\mathrm{E}_{\boldsymbol{\theta}}\left[{\lim}_{M\rightarrow \infty}\mathrm{E}\left[\sum_{t=0}^{M-1}\zeta^{t} r\left(\mathbf{X}(t),\mathbf{X}(t+1),\mu_{t}(\mathbf{X}(t)\right)\! \left|{\vphantom{\sum}} X_{0}\right.\right]\right]  \\ &{}={\lim}_{M\rightarrow \infty}\sum_{t=0}^{M-1}\mathrm{E}^{\ast}_{\boldsymbol{\theta}}\left[\zeta^{t} r\left(\mathbf{X}(t),\mathbf{X}(t+1),\mu_{t}(\mathbf{X}(t)\right)\! \left|{\vphantom{\sum}} X_{0}\right.\right],  \end{array} $$

where $\mathrm {E}^{\ast }_{\boldsymbol {\theta }}[\!\cdot ]=\mathrm {E}_{\boldsymbol {\theta }}[\mathrm {E}[\![\!\cdot ]\!]$ is the expectation over both within-model stochasticity and model uncertainty. For initial state *X*_0_, the optimal average cost is defined as

21 $$\begin{array}{*{20}l} J^{\Theta}(X_{0})=\underset{\mu\in\Pi}{\min}\,J^{\Theta}(\mu;X_{0}), \end{array} $$

and the minimum is attained by the IBR control policy $\mu ^{\Theta }(X_{0})=\left (\mu ^{\Theta }_{1}(X_{0}),\mu _{2}^{\Theta }(X_{0}),\dots \right)$.

This control problem can be transformed into a dynamic programming problem of the following form for each $i\in \mathcal {S}$ and *t*≥0:

22 $$\begin{array}{*{20}l} J_{t}(i)&=\underset{c\in\mathcal{C}}{\min}\,\mathrm{E}_{\boldsymbol{\theta}}\left[\mathrm{E}\left[r(i,j,c)+\zeta J_{t+1}(j)\right]\right] \notag \\ &=\underset{c\in\mathcal{C}}{\min}\,\mathrm{E}_{\boldsymbol{\theta}}\left[\sum\limits_{j=0}^{2^{n}-1}P^{\boldsymbol{\theta}}_{ij}(c)\left(r(i,j,c)+\zeta J_{t+1}(j)\right)\right] \notag \\ &=\underset{c\in\mathcal{C}}{\min}\left[\sum\limits_{j=0}^{2^{n}-1}\mathrm{E}_{\boldsymbol{\theta}}\left[P^{\boldsymbol{\theta}}_{ij}(c)\right]\left(r(i,j,c)+\zeta J_{t+1}(j)\right)\right].  \end{array} $$

We call $\mathrm {E}_{\boldsymbol {\theta }}\left [P^{\boldsymbol {\theta }}_{ij}(c)\right ]$ the *effective controlled transition probability matrix*. It is obtained similarly to *P*_*ij*_(*c*) by plugging $P_{ij}^{\Theta }=\mathrm {E}_{\boldsymbol {\theta }}\left [P_{ij}^{\boldsymbol {\theta }}\right ]$ in (4). The *effective transition probability matrix* (ETPM) $\mathbf {P}^{\Theta }=\left [P_{i,j}^{\Theta }\right ]_{i,j=1}^{2^{n}}$ is obtained as

23 $$\begin{array}{*{20}l} P_{ij}^{\Theta}=\mathrm{E}_{\boldsymbol{\theta}}\left[P_{ij}^{\boldsymbol{\theta}}\right]=\prod_{k=1}^{n}\mathrm{E}_{\boldsymbol{\theta}}\left[\text{Pr}_{\boldsymbol{\theta}}\left[X_{k}=j_{k}\left|\Gamma_{X_{k}}\right.[i]\right]\right],  \end{array} $$

where Pr_***θ***_[ ·] is the probability operator relative to ***θ*** and E_***θ***_[ ·] is taken relative to the updated prior (posterior) distribution $f(\boldsymbol {\theta }|\mathcal {X}_{L})$. The ETPM **P**^*Θ*^ represents the uncertainty class of TPMs and enables finding the IBR control policy *μ*^*Θ*^ for an uncertainty class of TPMs in the same way that the optimal control policy for a known TPM is found. Since the *θ*_*i*_’s are independent, the expectation can be brought inside the product. Each conditional probability term in (23) is either known, whose known value is used for multiplication, or is unknown and corresponds to an unknown parameter *θ*_*i*_ whose expected value obtained according to (19) is used in the multiplication.

The dynamic formulation in (22) is similar to the dynamic programming used for optimal control except that the known TPM has been replaced by $\mathrm {E}_{\boldsymbol {\theta }}\left [P^{\boldsymbol {\theta }}_{ij}(c)\right ]$; therefore, a similar approach used for solving optimal control dynamic programming can be utilized here with the distinction that all theorems should be defined relative to ETPM. Keeping this in mind, we define a mapping $TJ\!:\!\mathcal {S}\!\rightarrow \! \mathcal {R}$ for a bounded function $J\!:\!\mathcal {S}\!\rightarrow \!\mathcal {R}$ and $\forall i\!\in \!\mathcal {S}$ as

24 $$\begin{array}{*{20}l} TJ(i)=\underset{c\in\mathcal{C}}{\min}\left[\sum_{j=0}^{2^{n}-1}\mathrm{E}_{\boldsymbol{\theta}}\left[P^{\boldsymbol{\theta}}_{ij}(c)\right]\left(r(i,j,c)+\zeta J(j)\right)\right]. \end{array} $$

The following three theorems, whose proofs are similar to those in [40] relative to a known TPM, lay out the theoretical foundation for finding the IBR control policy.

#### **Theorem 1**

*(Convergence of the algorithm)* Letting $J:\mathcal {S}\rightarrow \mathcal {R}$ be a bounded function, for any $i\in \mathcal {S}$, the optimal average cost function *J*^*Θ*^(*i*) satisfies $J^{\Theta }(i)={\lim }_{M\rightarrow \infty }T^{M}J(i)\phantom {\dot {i}\!}$.

#### **Theorem 2**

*(Bellman’s optimality equation)* The optimal average cost function *J*^*Θ*^ satisfies

25 $$\begin{array}{*{20}l} {}J^{\Theta}(i)=\underset{c\in\mathcal{C}}{\min}\left[\sum\limits_{j=0}^{2^{n}-1}\mathrm{E}_{\boldsymbol{\theta}}\left[ P^{\boldsymbol{\theta}}_{ij}(c)\right]\left(r(i,j,c)+\zeta J^{\Theta}(j)\right)\right]\,\forall i\in\mathcal{S}.  \end{array} $$

#### **Theorem 3**

*(Necessary and sufficient condition)* A stationary policy *μ*^*Θ*^ is an IBR control policy if and only if for each $i\in \mathcal {S}$, *μ*^*Θ*^(*i*) attains the minimum in Bellman’s optimality equation.

Based on Theorem 1, *J*^*Θ*^ can be computed recursively using the value iteration algorithm in the same way that this algorithm is used to find the optimal control policy for a known TPM. The converged cost satisfies Bellman’s optimality equation (Theorem 2). Also, the corresponding policy is a stationary IBR control policy (Theorem 3). *μ*^*Θ*^ attains the minimum in the Bellman’s optimality equation, where the ordinary TPM is replaced by the ETPM.

The concept of effective quantities has also been used for deriving IBR operators in other problems: for example in [7], effective noise statistics are used to derive the IBR Kalman filter; or in [8], effective covariance matrix is used for achieving IBR signal compression.

To define the experimental design problem in the context of the framework laid out in (8)-(14), let the class of interventions *Ψ* be the set of all admissible control policies *Π*. Each *ψ*∈*Ψ* is characterized by a control policy *μ* and the cost of intervention is

26 $$\begin{array}{*{20}l} \xi_{\boldsymbol{\theta}}(\psi)=\mathrm{E}_{X_{0}}\left[J^{\boldsymbol{\theta}}_{\mu}(X_{0})\right],  \end{array} $$

where $\phantom {\dot {i}\!}J^{\boldsymbol {\theta }}_{\mu }(X_{0})$ is obtained according to (5) with E[ ·] relative to the probability measure defined by the TPM **P**^***θ***^. To find a single value as the cost of a control policy for a specific TPM, in (26), we take the expectation over all possible initial network states *X*_0_, assuming that the possible initial states are equally likely. Regarding the IBR intervention, we find a single value as the average cost of the IBR intervention:

27 $$\begin{array}{*{20}l} \mathrm{E}_{\boldsymbol{\theta}}\left[\xi_{\boldsymbol{\theta}}\left(\mu^{\Theta}\right)\right]=\mathrm{E}_{X_{0}}\left[J^{\Theta}(X_{0})\right],  \end{array} $$

where *J*^*Θ*^(*X*_0_) is obtained using (25). The definitions of cost and intervention in (26) and (27) set the stage for objective-based uncertainty quantification in the context of dynamical control according to (10). After defining MOCU, the MOCU-based experimental design framework can be used and the the primary parameter $\phantom {\dot {i}\!}\theta _{i^{\ast }}$ can be found by plugging (27) in (14):

28 $$\begin{array}{*{20}l} i^{\ast}&=\underset{}{\arg}\,\underset{i\in\{1,2,..,T\}}{\min}\,\mathrm{E}_{\theta_{i}^{\prime}}\left[\mathrm{E}_{\boldsymbol{\theta}|\theta^{\prime}_{i}}\left[\xi_{\boldsymbol{\theta}}\left(\psi_{\text{IBR}}\left(\Theta|\theta_{i}=\theta^{\prime}_{i}\right)\right)\right]\right] \notag \\ &=\underset{}{\arg}\,\underset{i\in\{1,2,..,T\}}{\min}\,\mathrm{E}_{\theta_{i}^{\prime}}\left[\xi_{\left(\Theta|\theta_{i}=\theta^{\prime}_{i}\right)}\left(\mu^{\Theta|\theta_{i}=\theta^{\prime}_{i}}\right)\right] \notag \\ &=\underset{}{\arg}\,\underset{i\in\{1,2,..,T\}}{\min}\,\mathrm{E}_{\theta_{i}^{\prime}}\left[\mathrm{E}_{X_{0}}\left[J^{\mathbf{P}^{\Theta|\theta_{i}=\theta^{\prime}_{i}}}_{\mu^{\Theta|\theta_{i}=\theta^{\prime}_{i}}}(X_{0})\right]\right],  \end{array} $$

where the IBR control policy for the reduced uncertainty class $\phantom {\dot {i}\!}\Theta |\left (\theta _{i}=\theta ^{\prime }_{i}\right)$ is found using the ETPM $\mathbf {P}^{\Theta |\theta _{i}=\theta ^{\prime }_{i}}\phantom {\dot {i}\!}$ obtained relative to the conditional probability distribution $f\left (\boldsymbol {\theta }|\theta _{i}=\theta ^{\prime }_{i}\right)\phantom {\dot {i}\!}$.

According to (28), to evaluate the determination of each unknown parameter *θ*_*i*_, for each realization $\theta ^{\prime }_{i}$ of *θ*_*i*_, we need to obtain the average cost of the IBR control policy $\mu ^{\Theta |\theta _{i}=\theta ^{\prime }_{i}}\phantom {\dot {i}\!}$ across the reduced uncertainty class $\Theta |\left (\theta _{i}=\theta ^{\prime }_{i}\right)\phantom {\dot {i}\!}$ and then take the average of all these average costs relative to the marginal distribution of parameter *θ*_*i*_. In practice, the expression in (28) is approximated via Monte-Carlo simulations. We draw a number of samples from the marginal distribution of *θ*_*i*_ and then approximate the expression being minimized in (28) as the average of all inner expectations computed for each generated sample. The steps required for obtaining the primary parameter $\phantom {\dot {i}\!}\theta _{i^{\ast }}$ are summarized in Algorithm 1. The inputs to this algorithm are *n* CPMs characterizing the GRN, *T* unknown parameters *θ*_*i*_ corresponding to unknown conditional probabilities, hyperparameters (*α*_*i*_,*β*_*i*_) for the prior beta distributions, the state trajectory $\mathcal {X}_{L}$, and *ζ*, *r*, and *I*, which determine the discount factor, cost function, and the number of iterations for value iteration, respectively.

Finding IBR control policies for an uncertainty class (like finding the optimal control policy for a known TPM [41]) is computationally expensive and the complexity grows exponentially with the number of genes. Therefore, most of the computational burden of the experimental design is in finding IBR control policies. To mitigate the complexity of the proposed method, in the next section we propose an approximate method for computing IBR control policies.

### Approximate experimental design based on MFPT

The mean first passage time (MFPT) from state *i* to *j* measures how long it would take on average that the network transitions from state *i* to state *j*.

For a Markovian GRN, if the sets of desirable states $\mathcal {D}$ and undesirable states $\mathcal {U}$ are determined, we can have the following partitioning for the TPM:

29 $$\begin{array}{*{20}l} \mathbf{P}= \left[\begin{array}{ll} \mathbf{P}_{\mathcal{D},\mathcal{D}} & \mathbf{P}_{\mathcal{D},\mathcal{U}}\\ \mathbf{P}_{\mathcal{U},\mathcal{D}} & \mathbf{P}_{\mathcal{U},\mathcal{U}} \end{array}\right],  \end{array} $$

where $\mathbf {P}_{\mathcal {S}_{1},\mathcal {S}_{2}}$ involves the transition probabilities from each state in the set $\mathcal {S}_{1}$ to the states in set $\mathcal {S}_{2}$. The vectors $\mathbf {K}_{\mathcal {D},\mathcal {U}}$ and $\mathbf {K}_{\mathcal {U},\mathcal {D}}$ of MFPTs from each state in $\mathcal {D}$ to $\mathcal {U}$ and from each state in $\mathcal {U}$ to $\mathcal {D}$, respectively, can be computed as [30]

30 $$\begin{array}{*{20}l} &\mathbf{K}_{\mathcal{D},\mathcal{U}}=\mathbf{e}+\mathbf{P}_{\mathcal{D},\mathcal{D}}\,\mathbf{K}_{\mathcal{D},\mathcal{U}}  \end{array} $$

31 $$\begin{array}{*{20}l} &\mathbf{K}_{\mathcal{U},\mathcal{D}}=\mathbf{e}+\mathbf{P}_{\mathcal{U},\mathcal{U}}\,\mathbf{K}_{\mathcal{U},\mathcal{D}},  \end{array} $$

where **e** is an all-unity column vector of appropriate size. If *g* is the control gene and $\tilde {X}^{g}$ is the flipped state corresponding to state *X* obtained by flipping *g* in state *X*, then to find the MFPT-based stationary control policy $\mu _{\mathbf {P}}^{\text {MFPT}}:\mathcal {S}\rightarrow \mathcal {C}$, the control action for each desirable state $X \in \mathcal {D}$ is obtained as [31]

32 $$\begin{array}{*{20}l} \mu^{\text{MFPT}}_{\mathbf{P}}(X)=\left\{ \begin{array}{l} 1\qquad \text{if}\,\,\mathbf{K}_{\mathcal{D},\mathcal{U}}(\tilde{X}^{g})-\mathbf{K}_{\mathcal{D},\mathcal{U}}(X)>\Delta \\ 0 \qquad \text{otherwise} \\ \end{array} \right.,  \end{array} $$

and for each undesirable state $X\in \mathcal {U}$ as

33 $$\begin{array}{*{20}l} {}\mu^{\text{MFPT}}_{\mathbf{P}}(X)=\left\{ \begin{array}{l} 1\qquad \text{if}\,\,\mathbf{K}_{\mathcal{U},\mathcal{D}}(X)-\mathbf{K}_{\mathcal{U},\mathcal{D}}(\tilde{X}^{g})>\Delta \\ 0\qquad \text{otherwise} \\ \end{array} \right.,  \end{array} $$

where *Δ* in (32) and (33) is a tuning parameter that should be adjusted based on the definition for the cost function *r*(*i*,*j*,*u*).

In the spirit of the MFPT-based approximation for optimal control, we approximate the IBR control policy needed in experimental design via MFPT. Taking into account that the IBR control policy *μ*^*Θ*^ is in fact the optimal control policy relative to the ETPM and that MFPT can be used as an approximation for the optimal control policy, we approximate the IBR control policy by finding the MFPT-based control policy relative to the ETPM and denote it by $\mu ^{\text {MFPT}}_{\Theta }$, i.e.,


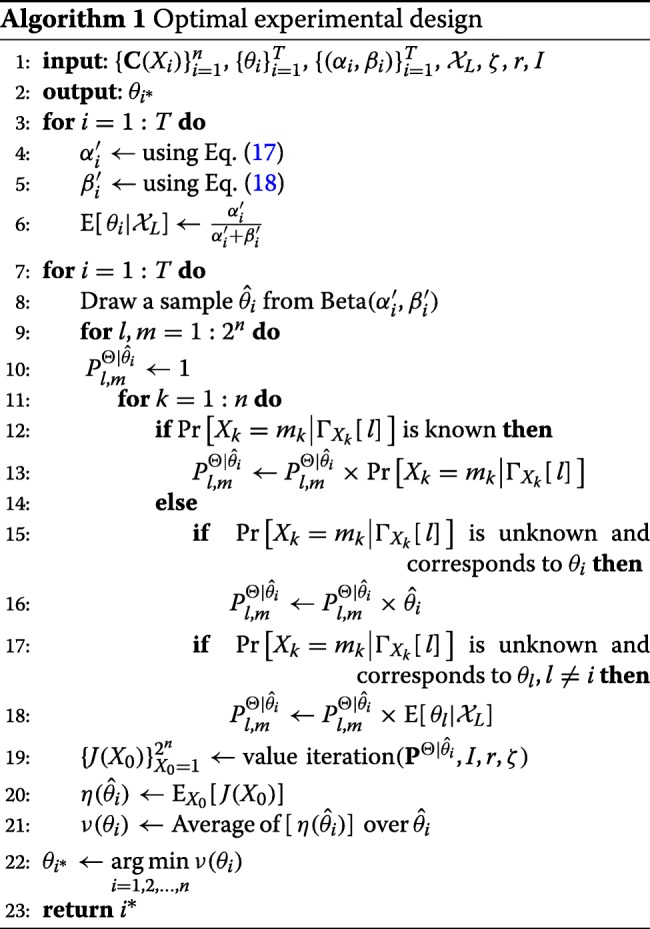

34 $$\begin{array}{*{20}l} \mu^{\Theta}\approx\mu^{\text{MFPT}}_{\Theta}=\mu^{\text{MFPT}}_{\mathbf{P}^{\Theta}}.  \end{array} $$

$\mu ^{\text {MFPT}}_{\mathbf {P}^{\Theta }}$ is obtained by solving (29)-(33) for the effective transition probability matrix **P**^*Θ*^. When we approximate the IBR control policy via MFPT in experimental design, the average cost needed in (28) is computed via Monte-Carlo simulations (over different initial network states) as

35 $$ {\begin{aligned} &\mathrm{E}_{X_{0}}\left[J^{\mathbf{P}^{\Theta|\theta_{i}=\theta^{\prime}_{i}}}_{\mu^{\Theta|\theta_{i}=\theta^{\prime}_{i}}}(X_{0})\right]\\ &\qquad\approx\ \frac{1}{N}\sum_{n=1}^{N}\left\{{\lim}_{M\rightarrow\infty}\sum_{t=0}^{M-1}\zeta^{t} r^{(n)}\left(\mathbf{X}(t),\mathbf{X}(t+1),\mu^{\text{MFPT}}_{\mathbf{P}^{{\Theta|\theta_{i}=\theta^{\prime}_{i}}}}(\mathbf{X}(t))\right)\right\},  \end{aligned}}  $$

where *N* is the total number of simulations and *r*^(*n*)^(·) is the accrued total discounted cost in the *n*-th simulation. The pseudo-code for the approximate method is the same as Algorithm 1 except for steps 19 and 20. For the approximate method, in step 19, we find $\mu ^{\text {MFPT}}_{\Theta |\hat {\theta }_{i}}$ via the MFPT approach. Then in step 20, we plug $\mu ^{\text {MFPT}}_{\Theta |\hat {\theta }_{i}}$, obtained from step 19, in (35) to compute $\eta (\hat {\theta }_{i})$.

Having used the MFPT approach for the sole purpose of reducing the uncertainty class, the IBR control policy is obtained by solving Bellman’s equation using value iteration.

### Computational complexity analysis

The computationally demanding step in the proposed experimental design method is to find IBR control policies. If there are *T* different unknown parameters and we generate *M* different samples for Monte-Carlo simulations for each one, then we need to find IBR control policies in our experimental design calculations *T*×*M* times. Since we use value iterations to solve Bellman’s equation, if we assume that the value iteration converges in *I* iterations, then we need to compute $(|\mathcal {S}|\times |\mathcal {C}|)^{I}$ terminal costs to obtain the control policy, $|\mathcal {S}|$ being the number of network states and $|\mathcal {C}|$ being the number of control actions. In this paper, we focus on binary networks and binary control actions, i.e., $|\mathcal {S}|=2^{n}$, *n* being the number of genes and $\mathcal {C}=2$. Therefore, the order of complexity when experimental design based on the IBR control policy is implemented is $\mathcal {O}\left (T\times M\times (2^{n+1})^{I}\right)$. The complexity grows exponentially with the number of genes and polynomially with the number of unknown parameters.

The complexity of the approximate experimental design is much lower because there is no iterative calculation in MFPT. It is enough to solve the two linear equations in (30) and (31), which involves two matrix inversions. Although applying MFPT for experimental design requires us to find the average cost of the MFPT-based IBR control policy via Monte-Carlo simulations, this overhead complexity for MFPT is not concerning and still the complexity of the MFPT-based approach is much smaller in comparison to that of the optimal experimental design. Since the calculations for each unknown parameter and each realization of that parameter can be done independently, a parallel implementation can be used for the proposed experimental design methods.

**Fig. 2:**
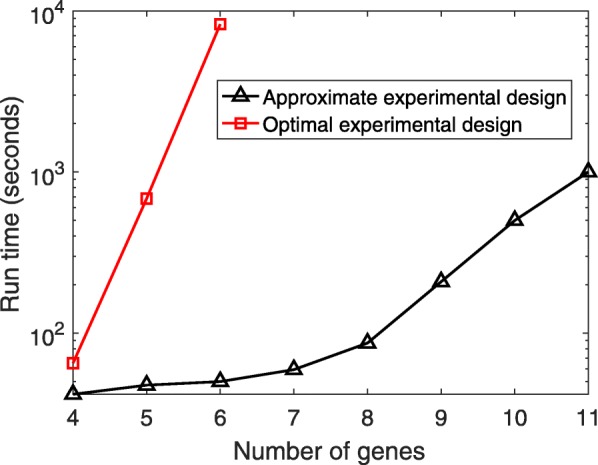
Approximate run time in seconds elapsed for the optimal (based on the value iteration method) and approximate experimental design methods (based on MFPT)

In Fig. 2, we provide run times required for finding the primary parameter among 5 unknown parameters for GRNs with different numbers of genes. The codes are scripted in MATLAB and run in a parallel framework on a Machine with an Intel^®^ quad-core 2.67 GHz CPU and 12 GB RAM. The number of iterations for value iteration is *I*=4. While the execution time grows exponentially with the number of genes and the runs may be prohibitively time-consuming beyond six genes for the optimal experimental design, the MFPT-based approximate method can be still implemented for networks of larger size.

## Results

In this section, we study the performance of the proposed methods based on synthetic and real gene networks. As a class of Markovian regulatory network, we consider Boolean networks with perturbation for the simulations.

### Boolean networks with perturbation

An *n*-gene Boolean network is defined by a set of binary variables *V*={*X*_1_,*X*_2_,..,*X*_*n*_}, and a set of Boolean functions **F**={*f*_1_,*f*_2_,…,*f*_*n*_}, where $f_{i}\!:\{0,1\}^{k_{i}}\rightarrow \{0,1\}$ determines the value of gene *X*_*i*_ when it has *k*_*i*_ regulating genes. The transition rule **X**(*t*+1)=**F**(**X**(*t*)) governs the evolution of states over time. In a Boolean network with perturbation (BNp), each gene may flip its value with a small perturbation probability *p*. In this network, the next state at time *t*+1 is **F**(**X**(*t*)) with probability (1−*p*)^*n*^ or **F**(**X**(*t*))⊕*γ* with probability 1−(1−*p*)^*n*^, where *γ* is a binary vector of size *n* and ⊕ is the component-wise addition modulo 2 operator. The underlying state evolution of a BNp over time can be viewed as a Markov chain with a transition probability matrix **P**. The TPM can be derived using the regulatory structure of the network and the perturbation probability [22]. When *p* > 0 the Markov chain is guaranteed to possess a unique steady-state distribution **π**.

### Synthetic networks

We first randomly generate a number of BNps and then from the corresponding TPMs we extract the set of conditional probabilities for each gene in the network. We consider BNps with 6 genes. The number of regulating genes for each gene is set to 2 and they are randomly selected from the set of genes. Therefore, the size of the CPM for each gene is 4×2. The bias (probability) that a Boolean function takes on the value 1 is randomly selected from a beta distribution with variance 0.0001 and mean 0.5. The perturbation probability *p* is set to 0.01. We use this protocol to generate 100 random BNps from which we generate 100 different sets of CPMs.

The conditional probability $\phantom {\dot {i}\!}\mathbf {C}_{j,1}(X_{i})\,=\,\text {Pr}[\!X_{i}\,=\,0|\Gamma _{X_{i}}=j]$ characterizing the regulation of gene *X*_*i*_ is obtained from the generated TPM **P** as

36 $$\begin{array}{*{20}l} \mathbf{C}_{j,1}(X_{i})=\sum\limits_{S^{\prime}}P_{SS^{\prime}},  \end{array} $$

where $\Gamma _{X_{i}}[\!S]=j$ and $S^{\prime }_{i}=0$. In other words, to find the conditional probability for gene *X*_*i*_ being down regulated when the equivalent decimal value of its regulating genes $\Gamma _{X_{i}}$ is *j*, we look for the row in the TPM in which $\Gamma _{X_{i}}$ is *j* and then in that row we take the summation of all TPM entries corresponding to gene *X*_*i*_ equal to 0. Similarly, we extract $\mathbf {C}_{j,2}(X_{i})=\text {Pr}[\!X_{i}=1|\Gamma _{X_{i}}=j]\phantom {\dot {i}\!}$ from the generated TPM as

37 $$\begin{array}{*{20}l} \mathbf{C}_{j,2}(X_{i})=\sum\limits_{S^{\prime}}P_{SS^{\prime}}, \end{array} $$

where $\Gamma _{X_{i}}[\!S]=j$ and $S^{\prime }_{i}=1$. Since more than one row in a TPM might correspond to $\Gamma _{X_{i}}=j$, in order to have a consistent procedure for extracting conditional probabilities, we take the average of all the values found for the rows corresponding to $\Gamma _{X_{i}}=j$.

To define the control problem, we assume that states with down-regulated genes *X*_1_ and *X*_2_ are undesirable, i.e., $\mathcal {U}=\{1,\dots,16\}$. The control gene whose expression can be flipped via control actions is *X*_6_. We use the following cost function for the simulations:

38 $$\begin{array}{*{20}l} r(i,j,c)=\left\{ \begin{array}{l} 6\qquad \qquad \text{if}\,\,j\in \mathcal{U}\quad \text{and}\quad c=1 \\ 5\qquad \qquad \text{if}\,\,j\in \mathcal{U} \quad\text{and}\quad c=0 \\ 1\qquad \qquad \text{if}\,\,j\in \mathcal{D}\quad \text{and}\quad c=1 \\ 0 \qquad \qquad \text{if}\,\,j\in \mathcal{D} \quad\text{and}\quad c=0 \end{array} \right..  \end{array} $$

This cost function reflects penalties assigned to undesirable states and also to the transitions to which the control action is applied. The discount factor *ζ* is set to 0.2. The tuning parameter *Δ* for the MFPT method is set to *Δ*=0.3. We use value iteration with 4 iterations to find the control policies in the optimal design method and for evaluating chosen experiments. All initial beta distributions for unknown conditional probabilities *θ*_*i*_ in the network are Beta(1,1), a uniform distribution. We run simulations for different numbers *L* of initial data used for updating priors.

In the first set of simulations, we generate 100 synthetic BNps. After extracting corresponding conditional probabilities for each network, we randomly select 5 conditional probabilities in each network and assume they are unknown. The aim is to decide which unknown conditional probability should be determined first. For each network, we generate a state trajectory $\mathcal {X}_{L}=\{\mathbf {X}(0),\dots,\mathbf {X}(L)\}$, used for updating initial hyperparameters, by simulating the underlying true network.

In the simulations, when we want to evaluate the determination of an unknown probability *θ*_*i*_, we put back its true value *ϕ*_*i*_, which was discarded during experimental design calculations, in the network, thereby resulting in a new uncertainty class *Θ*|(*θ*_*i*_=*ϕ*_*i*_) of remaining unknown probabilities. We find the IBR control policy $\mu ^{\Theta |(\theta _{i}=\phi _{i})}\phantom {\dot {i}\!}$ for this new uncertainty class by solving Bellman’s equation relative to $\mathbf {P}^{\Theta |(\theta _{i}=\phi _{i})}\phantom {\dot {i}\!}$. We then apply $\phantom {\dot {i}\!}\mu ^{\Theta |(\theta _{i}=\phi _{i})}$ to the underlying true network and run the controlled network until the horizon length 6 according to the underlying true TPM and $\mu ^{\Theta |(\theta _{i}=\phi _{i})}\phantom {\dot {i}\!}$, and record the cost at each time based on the network state at that time and the cost function *r*(*i*,*j*,*c*) in (38). Then we compute the total discounted cost over the horizon by accumulating the costs incurred over the horizon length according to the discount factor *ζ*. We repeat this process of calculating the total discounted cost for 10,000 iterations over different network initial states *X*_0_ and state transition paths. Note that although the underlying controlled TPM is fixed, there are still different state transition paths over the horizon due to the randomness characterized by the TPM. We represent the cost corresponding to determining parameter *θ*_*i*_ as the average of all 10,000 total discounted costs and denote it by *J*(*θ*_*i*_). For comparing different experimental design approaches, we report the average of *J*(*θ*_*i*_) over 100 generated synthetic networks and 100 different sets of assumed true values for the unknown probabilities in each network drawn from the beta prior distributions.

Using either optimal or approximate experimental design methods we can rank potential experiments $\mathcal {E}_{1}$ up to $\mathcal {E}_{5}$ from the optimal experiment being denoted by $\mathcal {E}_{1^{\prime }}$ (obtained according to (28)) to the least optimal experiment denoted by $\mathcal {E}_{5^{\prime }}$, which corresponds to the maximum value of the expression being minimized in (28). In Table 1, for different lengths *L* of the trajectory data used for updating priors, we rank experiments based on both experimental design methods and show the average cost $J(\theta _{i^{\prime }})$, 1≤*i*≤5, obtained after conducting experiment $\mathcal {E}_{i^{\prime }}$. This table suggests that the average cost obtained after conducting experiments with higher priority is smaller. Also, although the approximate experimental design method based on MFPT has much lower complexity, its performance is close to that of the optimal method. Note that the average cost obtained after high ranked experiments is lower when they are chosen by the optimal method but as we go towards low priority experiments the performance of the approximate method becomes better. This is because the optimal method yields a better ranking compared to the approximate method and more experiments resulting in lower average cost are given high priority in the optimal method. Another observation from the table is that, as we use more data for updating prior distributions, the difference between the performances of the different experiments gets smaller. For example, the difference between the average costs of $\mathcal {E}_{1^{\prime }}$ and $\mathcal {E}_{5^{\prime }}$ is larger when no data are used for the prior update than the case that the initial data $\mathcal {X}_{L}$ of length *L*=50 are used for the prior update. This is because by using more data in the prior update step the posterior distribution becomes tighter around the true model and less uncertainty remains in the model.

**Table 1:** Comparison of the ranked experiments according to the optimal and approximate methods

	$\mathcal {E}_{1^{\prime }}$	$\mathcal {E}_{2^{\prime }}$	$\mathcal {E}_{3^{\prime }}$	$\mathcal {E}_{4^{\prime }}$	$\mathcal {E}_{5^{\prime }}$
(a) *L*=0 (no initial data)					
Optimal	1.2215	1.2305	1.2399	1.2433	1.2427
Approximate	1.2246	1.2340	1.2388	1.2390	1.2416
(b) *L*=10					
Optimal	1.1615	1.1736	1.1780	1.1790	1.1792
Approximate	1.1646	1.1747	1.1767	1.1775	1.1779
(c) *L*=20					
Optimal	1.1573	1.1660	1.1706	1.1720	1.1705
Approximate	1.1598	1.1665	1.1701	1.1704	1.1695
(d) *L*=50					
Optimal	1.1463	1.1534	1.1558	1.1560	1.1561
Approximate	1.1487	1.1529	1.1557	1.1547	1.1557

Let *J*(***θ***_opt_), *J*(***θ***_approx_), and *J*(***θ***_rnd_) be the costs corresponding to the determination of the unknown probability chosen by the optimal method, the approximate method, and randomly, respectively. Table 2 shows the average of these costs over different networks and assumed true values. For different *L*, both optimal and approximate methods provide close performance and clearly outperform the random selection policy.

**Table 2:** The comparison of the average costs obtained after choosing the experiment via different selection policies

	*L*=0	*L*=10	*L*=20	*L*=50
*J*(***θ***_rnd_)	1.2350	1.1743	1.1673	1.1535
*J*(***θ***_approx_)	1.2246	1.1646	1.1598	1.1487
*J*(***θ***_opt_)	1.2215	1.1615	1.1573	1.1463

When comparing the optimal experiment $\mathcal {E}_{1^{\prime }}$ with an experiment $\mathcal {E}_{i^{\prime }}$, *i*≠1 (when experiments are ranked based on either optimal or approximate method), we say that a success occurs if $J(\theta _{1^{\prime }})-J(\theta _{i^{\prime }})<-0.002$, a failure happens if $J(\theta _{1^{\prime }})-J(\theta _{i^{\prime }})>0.002$, and a tie corresponds to $|J(\theta _{1^{\prime }})-J(\theta _{i^{\prime }})|<0.002$. Table 3 shows the ratio of success, failure, and tie for both methods and different *L*. Regardless of the experimental design approach, the ratio of success is always higher than the ratio of failure and gets larger when we compare the optimal experiment $\mathcal {E}_{1^{\prime }}$ with lowest ranked experiments. Note that the ratio of tie increases for larger values of *L* because a tighter prior leads to closer experimental performance.

**Table 3:** The percentage of success, failure, and tie for performing the chosen experiment rather than the suboptimal experiments

	$\mathcal {E}_{1^{\prime }}\sim \mathcal {E}_{2^{\prime }} $			$\mathcal {E}_{1^{\prime }}\sim \mathcal {E}_{3^{\prime }} $			$\mathcal {E}_{1^{\prime }}\sim \mathcal {E}_{4^{\prime }} $			$\mathcal {E}_{1^{\prime }}\sim \mathcal {E}_{5^{\prime }} $		
	S	F	T	S	F	T	S	F	T	S	F	T
(a) *L*=0 (no initial data)												
Optimal	49.8	45.3	4.9	54.9	39.8	5.3	55.2	39.6	5.1	55.9	38.7	5.4
Approximate	50.0	44.6	5.4	51.0	43.4	5.6	51.8	42.4	5.8	52.1	42.5	5.4
(b) *L*=10												
Optimal	54.0	40.9	5.1	55.3	38.6	6.0	56.4	37.8	5.8	56.8	37.7	5.5
Approximate	50.5	43.2	6.3	52.0	42.5	5.5	53	41.0	6.0	52.8	41.0	6.2
(c) *L*=20												
Optimal	50.4	43.8	5.8	52.1	41.5	6.4	52.8	41.4	5.8	53.9	40.3	5.7
Approximate	50.0	44.2	5.8	50.8	42.4	6.8	51.2	42.8	6.0	51.4	42.3	6.3
(d) *L*=50												
Optimal	50.1	43.2	6.7	52.2	41.2	6.6	52.9	40.0	7.1	52.3	41.8	5.9
Approximate	48.7	44.5	6.8	51.0	41.7	7.3	50.0	43.6	6.4	50.8	42.9	6.3

**Fig. 3:**
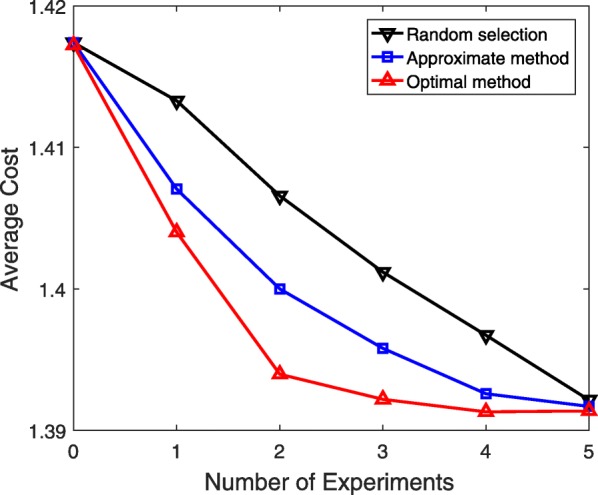
Performance evaluation of different experimental design approaches for a sequence of experiments. The size of initial data used for updating priors is *L*=0

Now, we evaluate the experimental design methods for a sequence of experiments. At each step in the sequential experiments, we choose experiment $\mathcal {E}_{i^{\ast }}$ based on the experimental design method. After incorporating the true value $\phantom {\dot {i}\!}\phi _{i^{\ast }}$ of the corresponding unknown probability $\theta _{i^{\ast }}\phantom {\dot {i}\!}$ in the model, we compute the cost $\phantom {\dot {i}\!}J(\theta _{i^{\ast }})$. The distribution for the new uncertainty class $\phantom {\dot {i}\!}\Theta |(\theta _{i^{\ast }}=\phi _{i^{\ast }})$ is the product of the beta distributions for the remaining unknown probabilities as we assume that all unknown probabilities are statistically independent. This distribution is used as the new prior distribution to find the next best experiment. This process continues until all unknown parameters are estimated and the underlying true network model is fully identified. Figure 3 presents the average cost over 50 different 6-gene networks and 100 different sets of assumed true values for optimal experimental design, approximate experimental design, and the random selection policy when there are *T*=5 unknown probabilities and no initial data is used for updating priors, i.e., *L*=0. Since the first data-point corresponds to the cost before any experiment and the final point corresponds to the cost after conducting all experiments, they are the same for all three curves. However, the decrease in the cost obtained by either experimental design method is faster in comparison to that of the random policy.

**Fig. 4:**
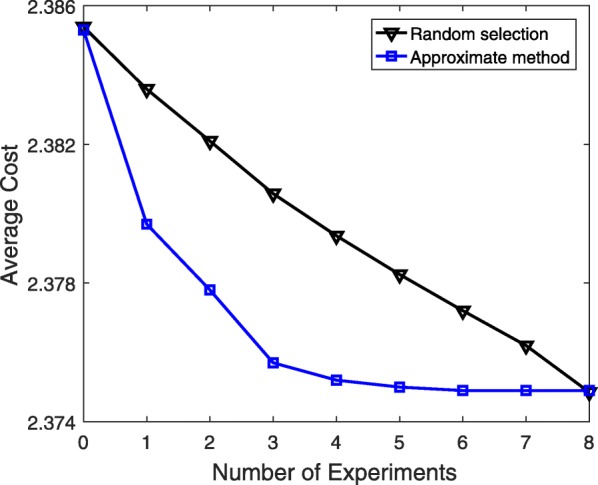
Performance evaluation of the approximate experimental design method and random selection policy for networks with 9 genes and 8 unknown probabilities. The length of $\mathcal {X}_{L}$ is *L*=5

Figure 4 compares approximate experimental design with the random selection policy when the network is of size *n*=9 and there are *T*=8 unknown probabilities. For this network size and number of unknown probabilities, the computational burden of optimal experimental design is prohibitively large. Therefore, we only implement the approximate method and the random selection policy. Recall that although we use the MFPT-based approximate approach for the experimental design step, the robust control policies after each experiment are still obtained by solving Bellman’s equation using value iteration. We see the promising performance of the approximate method in this figure. By following the approximate method, after conducting only four experiments the optimal cost is almost reached.

### Real network example: TP53 pathways

**Fig. 5:**
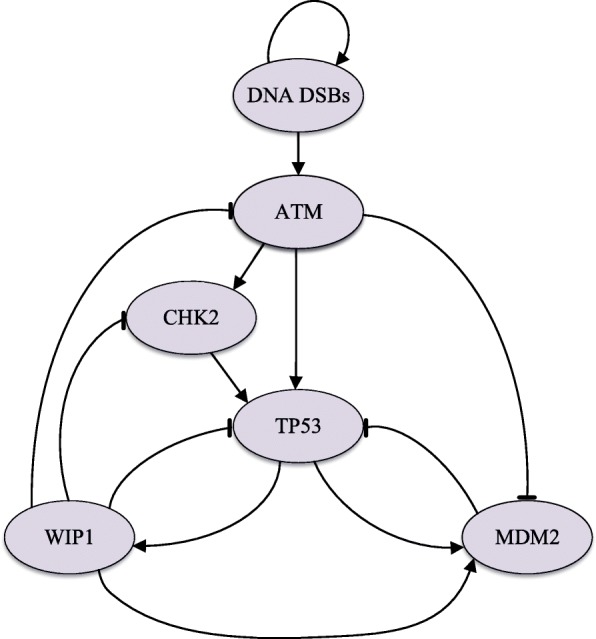
Regulatory relationships between genes in a signal pathway regulating the TP53 gene [42]

In this section, we consider the set of pathways involving the TP53 gene as shown in Fig. 5 [42]. TP53 is a tumor suppressor playing a major role in cellular activities in response to stress signals such as DNA damage. When DNA damage occurs, a mutant TP53 may lead to the abundance of abnormal cells, which eventually results in tumors. For example, it has been observed that mutated TP53 is present in 30 to 50% of human cancers [43]. In normal conditions, TP53 remains low-expressed under the control of MDM2, which is an oncogene often highly expressed in tumor cells. We model the pathways shown in Fig. 5 via a BNp with perturbation probability *p*=0.01. Six nodes DNA DSBs, MDM2, TP53, WIP1, CHK2, and ATM are named *X*_1_ up to *X*_6_, respectively. DNA DSBs is a signal that indicates the existence of double stand breaks. The dynamics of the network are governed via majority vote rule for which a regulatory matrix **R** defining the regulatory interactions between genes is defined as

39 $$ R_{ij}=\left\{ \begin{array}{l} \,\,\,\,1\quad \text{activating relation from}\,\,j\,\,\text{to}\,\,i \\ -1\quad \text{suppressive relation from}\,\,j\,\,\text{to}\,\,i \\ \,\,\,\,0\quad \text{no relation from}\,\,j\,\,\text{to}\,\,i \end{array} \right..  $$

Using matrix **R**, the value of gene *X*_*i*_ is updated as

40 $$ {}X_{i}(t+1)=f_{i}\left(\mathbf{X}(t)\right)=\left\{ \begin{array}{l} \,\,\,\,\,1\qquad \text{if}\,\,{\sum\nolimits}_{j}R_{ij}X_{j}(t)>0 \\ \,\,\,\,\,0\qquad \text{if}\,\,{\sum\nolimits}_{j}R_{ij}X_{j}(t)<0 \\ X_{i}(t)\qquad\!\!\!\!\!\! \text{if}\,\,{\sum\nolimits}_{j}R_{ij}X_{j}(t)=0 \end{array} \right.  $$

In Fig. 5, blunt arrows represent suppressive regulations and normal arrows represent activating regulations. It has been observed that in the presence of DNA damage (*X*_1_=1), up-regulated MDM2 (*X*_2_=1) and down-regulated TP53 (*X*_3_=0) would lead to cancerous cells [15, 44, 45]. Therefore, the set of undesirable states $\mathcal {U}$ includes those states with *X*_1_=0, *X*_2_=1, and *X*_3_=0, i.e., $\mathcal {U}=\{48,\dots,55\}$. The cost function *r*(*i*,*j*,*c*) is the same as the one given in (38). We also use gene ATM as the control gene.

**Fig. 6:**
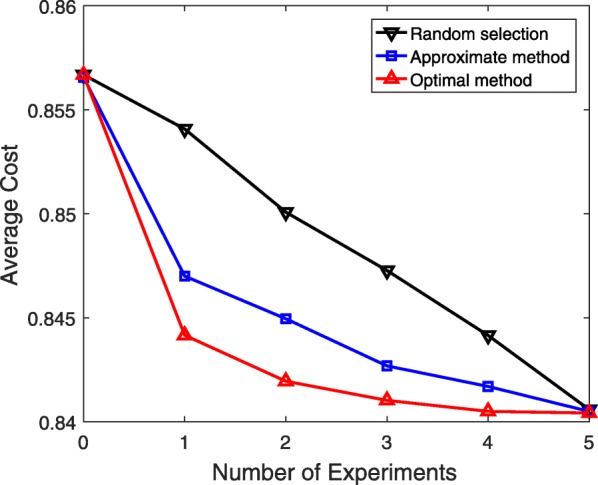
Performance evaluation of different experimental design approaches for a sequence of experiments based on the TP53 regulatory model

After building the BNp model from pathways, we extract the CPMs based on the procedure explained for simulations on synthetic networks. Since the network is fixed in this example, we randomly select 10 different sets of 5 conditional probabilities and assume that they are unknown. We run the experimental design simulations for 100 different assumed true values for each set of unknown probabilities. The length of $\mathcal {X}_{L}$ used for updating beta priors is *L*=5. Figure 6 illustrates the average cost obtained (over 1,000 different simulations) after each experiment in a sequence of experiments when optimal experimental design, approximate experimental design, or the random selection is employed. Better performance of both proposed approaches in comparison to the random selection policy is obvious from this.

### Real network example: mammalian cell cycle

As another example of real gene regulatory networks, consider the 9-gene mutated cell cycle network model. Cell cycle is a tightly controlled process initiated only in response to external stimuli, such as growth factors, under normal situations. A regulatory model containing 10 genes is proposed for the normal cell cycle in [46]. These 10 genes are the genes in Table 4 along with gene p27. A permanently down-regulated gene p27 in the cell cycle network results in a mutated cell cycle network consisting of 9 genes. The Boolean functions for the mutated network are summarized in Table 4 [46]. We use these Boolean functions and build a BNp model with perturbation probability *p*=0.01. The index of each gene in the BNp model is given in the table. In this network, if both Cyclin D (CycD) and retinoblastoma (Rb) are down-regulated, then cell cycle continues even in the absence of stimuli, thereby leading to the growth of tumors. Hence, states with down-regulated CycD (*X*_1_=0) and down-regulated Rb (*X*_2_=0) are undesirable. To define a control problem, we use the cost function in (38) and choose gene CycA as the control gene.

**Table 4:** The set of Boolean functions for a mutated cell cycle [46]

Gene	Node	Boolean function
CycD	*X*_1_	Extracellular signal
Rb	*X*_2_	$(\overline {X_{1}}\wedge \overline {X_{4}}\wedge \overline {X_{9}}\wedge \overline {X_{8}})$
E2F	*X*_3_	$(\overline {X_{2}}\wedge \overline {X_{9}}\wedge \overline {X_{8}})$
CycE	*X*_4_	$(X_{3}\wedge \overline {X_{2}})$
Cdc20	*X*_5_	*X*_8_
Cdh1	*X*_6_	$(\overline {X_{9}}\wedge \overline {X_{8}})\vee X_{5}$
UbcH10	*X*_7_	$\overline {X_{6}}\vee (X_{6}\wedge X_{7} \wedge (X_{5}\vee X_{9}\vee X_{8}))$
CycB	*X*_8_	$(\overline {X_{5}}\wedge \overline {X_{6}})$
CycA	*X*_9_	$(X_{3}\wedge \overline {X_{2}}\wedge \overline {X_{5}}\wedge (\overline {X_{6}}\wedge \overline {X_{7}}))$
		$\vee (X_{9}\wedge \overline {X_{2}}\wedge \overline {X_{5}}\wedge (\overline {X_{6}\wedge X_{7}}))$

**Fig. 7:**
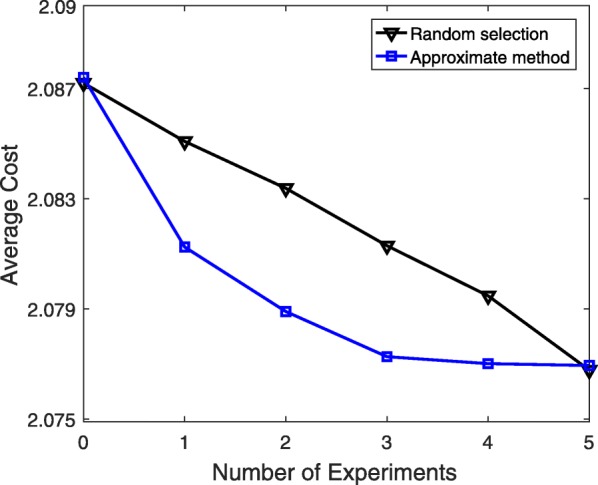
Performance evaluation of the approximate experimental design for a sequence of experiments based on the mutated mammalian cell cycle model

Due to the size of the mammalian cell cycle, the optimal experimental design is not applicable. Therefore, for this network, we compare the approximate method and the random selection policies. The simulation settings are exactly the same as those for the TP53 model. We generate a state trajectory of size *L*=5 for updating priors. Simulation results in Fig. 7 are averaged over 10 different selections of sets of 5 unknown probabilities and 100 different assumed true values for each. The promising performance of the approximate method is clear in this figure.

## Discussion

An inherent problem of dealing with stationary control policies in a Markovian network is computational complexity, which is due to the exponential increase of the number of states with the network size. We have been able to mitigate the complexity of the experimental design and thereby push the size limit (as demonstrated in Fig. 2) by proposing an approximate experimental design approach based on mean first passage time. However, we believe that more complexity reduction should be achieved for addressing experimental design for extremely large gene networks. Considering these intrinsic computational issues, we plan to find more efficient approximations and investigate accelerated implementation of the method via efficient computer architecture platforms, such as Graphic Processing Unit (GPU).

Another consideration is the accuracy of the prior distributions used in the experimental design calculation. The performance of the experimental design depends on the degree to which the prior probabilities can describe the existing knowledge regarding uncertain parameters accurately. The problem of finding optimal prior probabilities in this context can be solved under a prior construction optimization framework, which involves constructing a mapping that transforms signaling relations to constraints on the prior distribution. Constructing optimal priors has been done for genomic classification [14, 47]. In future work, we aim to develop an optimization framework to address prior construction for experimental design in gene regulatory networks.

## Conclusions

Given the complexity of biological systems and the cost of experiments, experimental design is of great practical significance in translational genomics. In this paper, we address the problem of optimal experimental design for gene regulatory networks controlled with stationary control policies. The proposed experimental design framework is based on the notion of mean objective cost of uncertainty, which views model uncertainty in terms of the increased cost it induces. Future work includes further reducing the computational cost of the method and also designing optimization frameworks for constructing optimal prior distributions. Also, another avenue of research is to implement an integrative experimental design method that can utilize the RNA-Seq data for the genes on the same pathway [48] for optimal uncertainty reduction.

## Abbreviations

BNp: Boolean network with perturbation
CPM: Conditional probability matrix
ETPM: Expected transition probability matrix
GRN: Gene regulatory network
IBR: Intrinsically Bayesian robust
MDP: Markov decision process
MFPT: Mean first passage time
MOCU: Mean objective cost of uncertainty
TPM: Transition probability matrix
